# Growth-Promoting Effects of Zhenqi Granules on Finishing Pigs

**DOI:** 10.3390/ani12243521

**Published:** 2022-12-13

**Authors:** Wentao Luo, Yaxue Huang, Xiuxiu Qiu, Wenxiao Zhuo, Yujun Tao, Shuaiyang Wang, Huaixia Li, Jing Shen, Lelin Zhao, Lijun Zhang, Shuo Li, Jie Liu, Qi Huang, Rui Zhou

**Affiliations:** 1 State Key Laboratory of Agricultural Microbiology, and Cooperative Innovation Center for Sustainable Pig Production, Huazhong Agricultural University College of Veterinary Medicine, Wuhan 430070, China; 2 Hubei Provincial Veterinary Drug Research Center, HVSEN Biotech, Wuhan 430042, China

**Keywords:** Zhenqi granules, growth promotion, carcass traits, transcriptomics, finishing pigs

## Abstract

**Simple Summary:**

With the ban on using antibiotics as animal growth promoters in many countries, developing alternative growth promoters is of urgent need. In this study, we investigated the growth-promoting efficacy of a traditional Chinese medicine (TCM) formula Zhenqi granules (ZQ) in pigs. We show that ZQ has a significant growth-promoting effect in pigs, especially during the finishing stage. To further explore the possible mechanisms of growth promotion, transcriptomics analysis with liver and skeletal muscle tissues are performed that reveals that genes involved in collagen biosynthesis and lipid biosynthetic processes are differentially expressed in the pigs administrated with ZQ. We report for the first time that a TCM formula ZQ has significant growth promotion efficacy in pigs and reveals that it may promote animal growth by regulating skeletal myogenesis and fat deposition.

**Abstract:**

Developing nonantibiotic livestock growth promoters attracts intensive interest in the post-antibiotic era. In this study, we investigated the growth-promoting efficacy of Zhenqi granules (ZQ) in pigs and further explored the possible mechanisms by transcriptomics analysis. Weaned piglets (52 days old with an average body weight of 17.92 kg) were fed with diets supplemented with different doses of ZQ (0 g/kg, 1 g/kg, and 2 g/kg) for 30 days and continued observations for an additional 32 days after removing ZQ from the diets. Compared with the control group, the average daily gain, carcass weight, average back fat thickness, and fat meat percentage of the group supplemented with 1 g/kg of ZQ showed a significant increase, and the feed/gain ratio was lower. The group supplemented with 2 g/kg of ZQ also showed a significant increase in average daily gain and average backfat thickness. A transcriptomics analysis revealed that the supplementation of ZQ at 1 g/kg upregulated the expression of genes related to collagen biosynthesis and lipid biosynthesis in skeletal muscle and liver. This effect was primarily through upregulating the mRNA levels of structural proteins and lipid-related enzymes. This study demonstrates the growth-promoting efficacy of ZQ and provides some insights of the mechanism of growth promotion.

## 1. Introduction

The demand for food products of animal origin is still increasing today with the growing global population. Thus, improving livestock performance is still an important need for intensive farming [1,2]. Previously, the application of subtherapeutic antibiotics as growth promoters in animal feed has made great achievements [3,4,5,6]. However, this also leads to a variety of serious antibiotic resistance problems around the world [7,8,9]. To address this threat, the use of antibiotics as growth promoters in food animal production has been forbidden in several countries [2], including China [10]. Due to high demand, in recent years, it has become a hot research topic to develop antibiotic alternatives as growth promoters. These include, but are not limited to, phytochemicals, acidifiers, probiotics, prebiotics, synbiotics, enzymes, bacteriophages, and antimicrobial peptides and probiotics, as well as probiotics, prebiotics, postbiotics, and parabiotics [2,11].

Traditional Chinese medicine (TCM) is a promising alternative to antibiotics with the characteristics of resource-rich, few side effects, multitargets, and multifunctional [12]. A few TCMs are demonstrated to be effective in promoting the growth of livestock [13,14,15]. For example, a study showed that dietary supplementation of Bazhen could significantly improve average daily gain (ADG) and feed efficiency (FE) by 4.73% and 17.9%, respectively, as well as decrease the average daily feed intake (ADFI) by 14.10% [16]. In addition, studies have suggested that extract [17], polysaccharides [18,19], and essential oils [20] of TCM also have a growth-promoting effect in pigs.

Zhenqi Granules (ZQ), a TCM formula, is composed of two TCMs, including Huangqi (*Astragalus membranaceus* (*Fisch.*)) and Nvzhenzi (*Fructus ligustri lucidi*). Modern pharmacologic studies have demonstrated that *Astragalus membranaceus* has immunomodulatory [21] and antiaging effects [22]. It has been proven that *Astragalus* polysaccharides (APS), the main ingredient of Huangqi, has the potential to improve livestock growth [23,24,25,26]. *Fructus ligustri lucidi* is a well-known invigorator, which contains several chemically active constituents, such as ursolic acid, oleanolic acids, oleanolic acid, and salidroside [27]. It has many pharmacological effects including hepatoprotective effect [28], antioxidant action [29], immunomodulatory effect [30], and therapeutic antiosteoporosis action [31].

Our previous results suggest that supplementation of ZQ in feed significantly reduced the feed to gain ratio (F/G) in finishing pigs [32]. However, it is still unknown whether supplemented with 1 (ZQ-1 g) and 2 g/kg of ZQ (ZQ-2 g) also has a growth-promoting efficacy for pigs in the early stage of farming. Moreover, the underlying mechanism of how ZQ promotes the growth of pigs remains largely unknown. Therefore, in this study, the growth-promoting efficacy was investigated by monitoring the growth performance of pigs supplemented with two doses of ZQ in the feed. Transcriptomics analysis was further carried out with liver and skeletal muscle tissues to compare the transcription changes by supplementation of ZQ.

## 2. Materials and Methods

### 2.1. Zhenqi Granules and Experimental Diets

Zhenqi Granules was provided by HVSEN Biotech Co., Ltd., Wuhan, China, of which 1 g of granules is equivalent to 0.5 g of the raw herbs. The animals were fed twice daily with standard commercial pig feed (Q/HJ 002-2020, Hubei Jinxinnong Feed Co., Ltd., Wuhan, China). The main ingredients of the experimental diets include corn, soybean meal, flour, whey powder, fish meal, soybean oil, vitamin A, vitamin D3, vitamin E, vitamin B2, choline chloride, manganese sulfate, copper sulfate, ferrous sulfate, zinc chloride, calcium hydrogen phosphate, stone powder, sodium chloride, and L-lysine hydrochloride. The ingredients and nutrient compositions of the diets are provided in Table 1.

### 2.2. Animals, Housing, and Experimental Design

**Figure 1:**
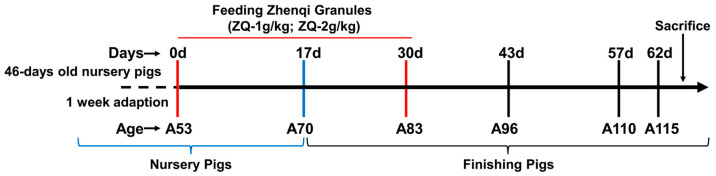
Schematic overview of the experimental design of the study. ZQ, Zhenqi granules. ZQ-1 g, 1 kg standard diet supplemented with 1 g ZQ. ZQ-2 g, 1 kg standard diet supplemented with 2 g ZQ. d, day from the beginning of the experiment. In the following, we use Day to indicate the days of the experiment. A, age of the pigs.

A total of 24 healthy castrated male Large White piglets (46 days old, A46) weighing 17.923 ± 2.462 kg were purchased from Tianzhong Animal Husbandry Co., Ltd., Wuhan, China. After adaptive feeding for a week (A53), the pigs were randomly distributed into 3 groups with 8 pigs in each. The two treatment groups were fed with basal diets supplemented with 1 (ZQ-1 g) and 2 g/kg of ZQ (ZQ-2 g), respectively, for 30 days. The pigs of the control group were just fed with basal diets. The experimental design is illustrated in Figure 1.

Pigs were housed in groups with 8 pigs in a pen. The temperature of pig houses was kept at 20–25 °C with rubber mats and heat lamps. The pens were cleaned once daily. The clinical health of the pigs was assessed daily. Two of the pigs showed stagnant growth at the early stage of the experiment for unknown reasons, so they were culled on Day 17. The experiment was carried out in the Experimental Pig Farm at the Breeding Swine Quality Supervision and Testing Center (Ministry of Agriculture and Rural Affairs) in Wuhan, China.

### 2.3. Samples and Data Collection

During the experiment, the feed intake was recorded daily. Meanwhile, the pigs were weighed, and blood samples were collected from the inferior vena cava on Day 0 (A53), Day 17 (A70), Day 30 (A83), Day 43 (A96), Day 57 (A110), and Day 62 (A115). The serum was separated from the blood by centrifugation at 1000× *g* for 20 min at room temperature and then stored at −80 °C to avoid repeated freezing and thawing until use.

### 2.4. Carcass and Meat Quality Traits

One month after the last administration (Day 62), five pigs from each group were slaughtered following fasting for 24 h and subjected to carcass and meat quality traits measurement. The carcass and meat quality traits analyzed in this study included live weight, carcass weight, dressing percentage, carcass length, average backfat thickness, loin eye area, leg percentage, skin percentage, fat meat percentage, lean meat percentage, bone percentage, meat marbling score, and intramuscular fat. All traits were measured by the Breeding Swine Quality Supervision and Testing Center according to the national profession standards (No. NY/T 821-2019, NY/T 1180-2006, NY/T 825-2004).

### 2.5. Serum GH and IGF-I

Serum GH and IGF-I were measured by enzyme-linked immunosorbent assay (ELISA) (Pig GH ELISA Kit, CSB-E06813p, Cusabio; Pig IGF-1 ELISA Kit, CSB-E06829p, Cusabio) according to the manufacturer’s instructions. Briefly, isolated swine serum, bio-antibody, and streptavidin-HRP were added into 96-well plates precoated with antibody and incubated at 37 °C, respectively. After 1 h, liquid was discarded and washed completely. Next, substrate solution was added into to each well incubated for 30 min at 37 °C in the dark. Finally, stop solution was added to each well, and absorbance was measured at 450 nm.

### 2.6. Biochemical Indexes of Blood

The serum biochemical indexes were detected by an automatic blood biochemical analyzer (BS-240; Shenzhen Mindray Bio-Medical Electronics Co., Ltd., Shenzhen, China). Creatinine (CREA) (lot#:141121008), glucose (GLU) (lot#:1415210016), alanine transaminase (ALT) (lot#:1401221003), aspartate aminotransferase (AST) (lot#:140220013), albumin (ALB) (lot#:148321001), triglyceride (TG) (lot#:141720010), total cholesterol (TCHO) (lot#:141621002), carbamide (UREA) (lot#:141321002), and total protein (TP) (lot#:140820006) assay kits were purchased from Shenzhen Mindray Bio-Medical Electronics Co., Ltd.

### 2.7. Transcriptome

The liver and skeletal muscle (longissimus dorsi) tissue samples were collected from 3 randomly chosen pigs from ZQ-1 g group and control group, respectively. The tissues were immediately frozen in liquid nitrogen and stored at −80 °C until use. The total RNA was extracted from the tissue using TRIzol^®^ Reagent (Invitrogen, Waltham, MA, USA). mRNA was isolated according to polyA selection method by oligo(dT) beads and reversely transcribed to cDNA by using a SuperScript double-stranded cDNA synthesis kit (Invitrogen, Waltham, MA, USA) with random hexamer primers (Illumina, San Diego, CA, USA). The samples were then subjected to high throughput sequencing using the Illumina HiSeq xten/NovaSeq 6000 (Illumina, San Diego, CA, USA) sequencer (2 × 150 bp read length) platform. The data were analyzed using the Majorbio Cloud Platform (www.majorbio.com) (accessed on 1 October 2022) [33].

### 2.8. Statistical Analysis

Statistical analysis was performed using SPSS software 16.0 (SPSS, Inc., Chicago, IL, USA). One-way ANOVA followed by LSD was used to compared the statistical differences among the three groups. Graphs were made using GraphPad Prism 9 (San Diego, CA, USA). All data were expressed as means ± standard deviation (SD). Differences with *p* < 0.05 and *p* < 0.01 were considered as significant and indicated as * and **, respectively. 

## 3. Results

### 3.1. Dietary Supplementation with ZQ Increases the Growth Performance of Pigs

**Figure 2:**
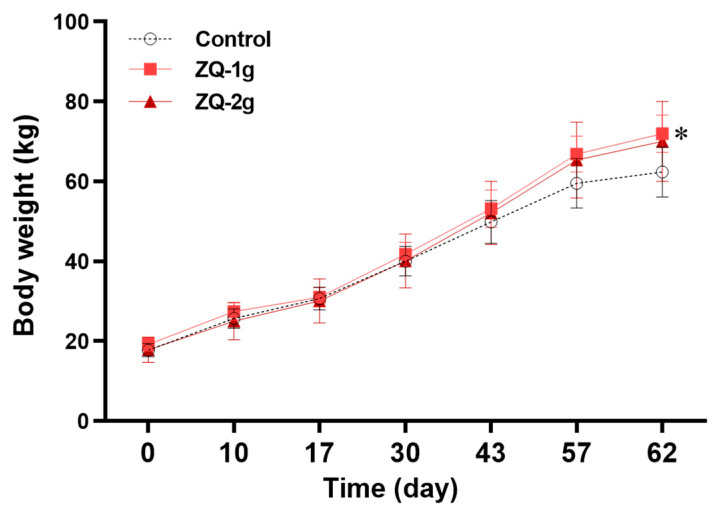
Growth curve of pigs. Note: * indicates a significant difference when the values were compared to that of the control (*p* < 0.05).

**Figure 3:**
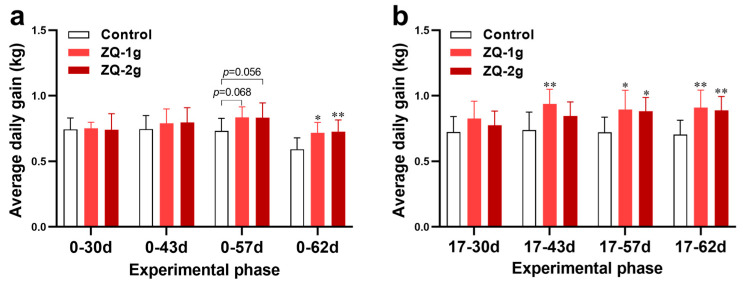
Effects of ZQ on ADG of pigs. (**a**) ADG at the nursery phase. (**b**) ADG at the finishing phase. * Indicates *p* < 0.05 and ** indicates *p* < 0.01 between the treatment group (ZQ-1 g or ZQ-2 g) and the control group.

In order to test the growth-promoting efficacy of ZQ, the body weight of pigs in each group was monitored. It was shown in Figure 2 that the initial average body weight was similar between each group without statistical significance. At the end of ZQ treatment (Day 30), there was still no significant difference in average body weight between ZQ-treated and control groups. Afterward, all three groups were fed normally without supplementation of ZQ. It was shown that the average body weight of ZQ-1 g group was significantly higher than that of the control group (*p* < 0.05) on Day 62. Although the ZQ-2 g group had a higher average body weight compared to the control group, it did not reach statistical significance (*p* = 0.056) (Figure 2, Table S1). Meanwhile, the ADG values of pigs in the two ZQ-treated groups over time during the experiment were both significantly higher than that of the control group (*p* < 0.05) (Figure 3a). A numerically smaller F/G was also observed for the ZQ-treated groups than for the control group (Table S2). 

The growing phase, which is usually from 70 days old (body weight of 30 kg), is the fastest growth phase across the life stages of pigs. Therefore, the growth performance of pigs was assessed from Day 17 (70 days old) to Day 62 (115 days old). The results revealed a significant increase in ADG of pigs in the ZQ-1 g group between 17 and 43 d compared to the control (*p* < 0.01). Similarly, the ADG in ZQ-1 g and ZQ-2 g pigs between 17 and 57 d was also significantly higher than that in the control (*p* < 0.05), and it showed a highly significant difference between Day 17 and Day 62 (*p* < 0.01) (Figure 3b). At this finishing phase, the F/G was lower in the ZQ-treated groups than that in the control group (Table 2). These data suggest that the addition of ZQ in the pig diets in the transition of the nursery pigs to the finishing pigs can effectively promote the growth of pigs.

### 3.2. Dietary Supplementation with ZQ Increases Carcass Weight and Fat Content of Pigs

**Figure 4:**
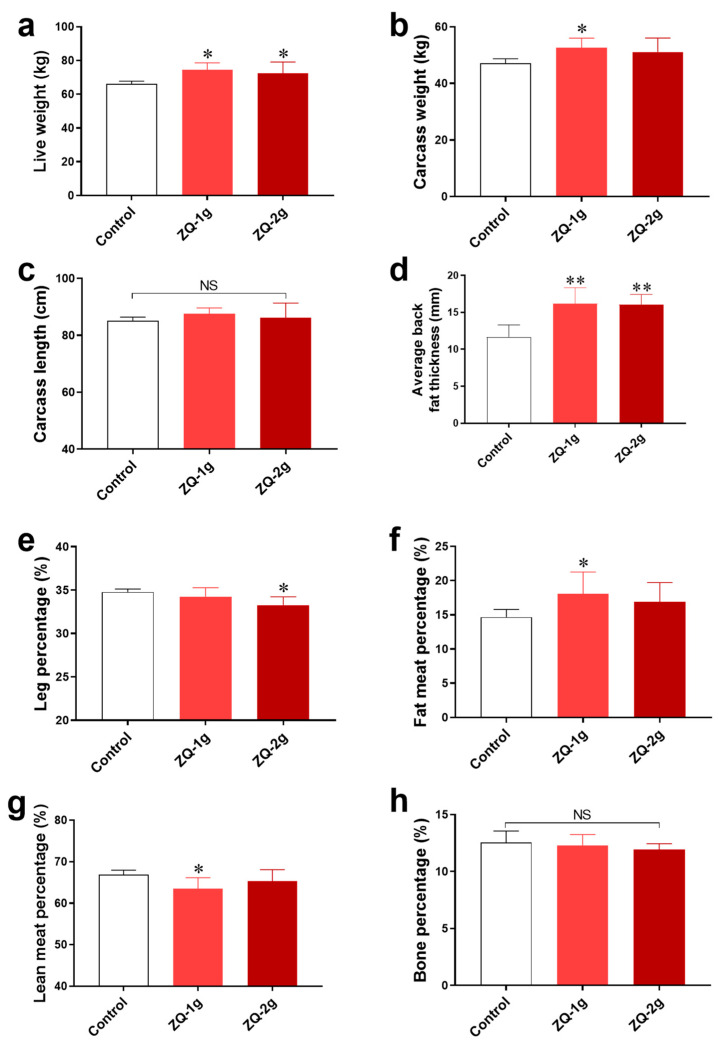
Effects of ZQ on carcass and meat quality traits in pigs. (**a**) Live weight. (**b**) Carcass weight. (**c**) Carcass length. (**d**) Average backfat thickness. (**e**) Leg percentage. (**f**) Fat meat percentage. (**g**) Lean meat percentage. (**h**) Bone percentage. Note: * indicates *p* < 0.05, ** indicates *p* < 0.01, and NS indicates no significant differences (*p* > 0.05).

To further confirm the effect of ZQ on growth performance, we determined the carcass and meat quality traits. As presented in Figure 4, pigs of ZQ-1 g group showed significantly higher live weight (Figure 4a), carcass weight (Figure 4b), average backfat thickness (Figure 4d), and fat meat percentage (Figure 4f) than the control group (*p* < 0.05). However, the lean meat percentage of ZQ-1 g group was lower (*p* < 0.05) (Figure 4g). Meanwhile, compared with the control group, the pigs in ZQ-2 g group also had a significant increase in live weight (Figure 4a) and average back fat thickness (*p* < 0.01) (Figure 4d) but had a significant decrease in the leg percentage (*p* < 0.05) (Figure 4e). There were no significant effects on carcass length (Figure 4c) and bone percentage (Figure 4h) due to the ZQ addition. These data indicate that ZQ may increase the fat and muscle content of finishing pigs. 

### 3.3. Effects of ZQ on Serum Hormones and Biochemical Indexes

We tested the indicators related to growth in pig serum. The concentrations of GH and IGF-I in serum were measured at the end of the experiment (Day 62), and the results showed that these hormones had similar levels in the ZQ-treated pigs and the control pigs (Table S3). After 30 days of feed supplementation with ZQ (Day 30), compared to the control group, UREA values of pigs in the ZQ-1 g were significantly increased (*p* < 0.05), and TG values in ZQ-2 g were significantly increased (*p* < 0.01). At the end of the experiment (Day 62), the levels of AST, TG, and CREA in the ZQ-1 g group were significantly lower than those in the control group (*p* < 0.05), but UREA was increased significantly (*p* < 0.05). The levels of Glu, TG, and CREA in the ZQ-2 g group were significantly lower than those in the control group (*p* < 0.05), but UREA was increased significantly (*p* < 0.01). However, it was worth noting that all the measured serum biochemical indices were in the normal range for all groups (Table 3). Therefore, the data suggest that ZQ does not exert growth promotion efficacy by directly increasing the level of growth hormones but possibly through metabolism regulation.

### 3.4. Transcriptome Analysis of Liver Tissues

**Figure 5:**
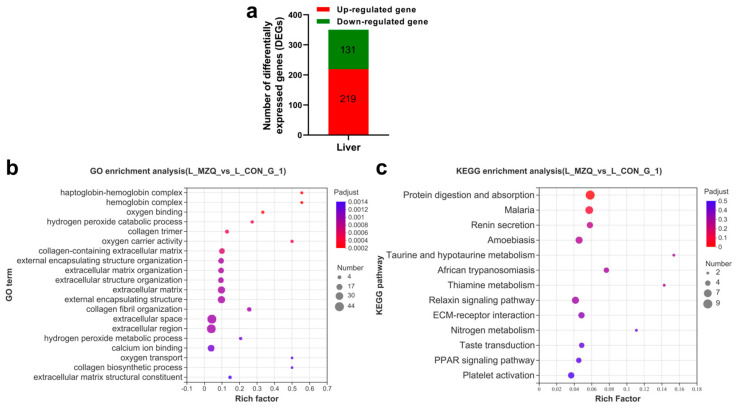
Transcriptomic analysis of the liver. (**a**) DEGs between the ZQ-1 g and control. (**b**) Bubble plot of the enriched GO terms of the liver. (**c**) Bubble plot of the enriched KEGG pathways of the liver.

As the liver is the key organ for metabolism, a transcriptome analysis was carried out with liver tissues to explore the molecular mechanisms underlying the phenotype. As illustrated in Figure 5a, 350 differentially expressed genes (DEGs) were found with 219 genes upregulated and 131 downregulated in liver tissues of pigs in the ZQ-1 g group compared with the control pigs (Figure 5a, Table S4). The KEGG pathway enrichment results revealed that the top-ranked pathways were protein digestion and absorption (Figure 5c, Table S6). In addition, GO enrichment showed that categories with collagen biosynthesis and the extracellular matrix (ECM), including collagen trimer, collagen-containing extracellular matrix, and extracellular matrix organization, were the most enriched ones (Figure 5b, Table S5).

**Figure 6:**
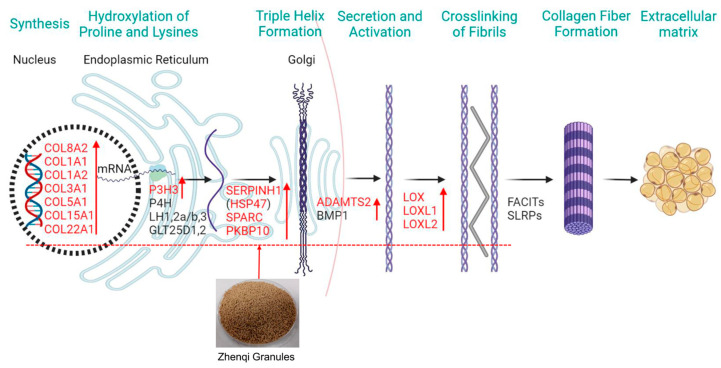
Collagen biosynthesis. Note: COL, collagen; P3H3, prolyl 3-hydroxylase 3; P4H, prolyl-4-hydroxylases; LH, lysyl hydroxylase; GLT25D, glycosyltransferase 25 domain containing 1; SERPINH1, serpin family H member 1; FKBP10, FKBP prolyl isomerase 10; SPARC, secreted protein acidic and cysteine rich; ADAMTS2, ADAM metallopeptidase with thrombospondin type 1 motif 2; HSP47, heat shock protein 47, also known as SERPINH1; BMP1, bone morphogenetic protein 1; LOX, lysyl oxidase; FACITs, fibril associated collagens with interrupted triple helices; SLRPs, small leucine-rich proteoglycans. Figure created with BioRender (San Francisco, CA, USA).

The biosynthesis of collagen is an extremely complex process [34] (Figure 6). First of all, the collagen mRNA was translated into nascent in the rough endoplasmic reticulum (rER). Next, the nascent collagen polypeptide chain is modified by glycosylation and prolyl-3-hydroxylation and folding of the C- and N-terminal propeptides. The mRNA of P3H3 which is an enzyme for post-translational modifications in the above process was upregulated in the ZQ-1 g group (Table 4). Then, the nascent collagen polypeptide chains assemble into triple helix formation in a zipper-like fashion and is dependent on SERPINH1(HSP47) which is a collagen chaperone, and the PPIases (peptidyl-prolyl isomerases) FKBP10 play critical roles in the linear prolongation of the triple helix. Interestingly, both the mRNA of SERPINH1 and FKBP10 were upregulated in the ZQ-1 g group (Table 4). Immediately following this, the collagen helices are secreted into the extracellular space via the trans-Golgi network. Extracellular processing and maturation of collagen are primarily related to ADAMTS protease family, and the mRNA of ADAMTS2 was also significantly upregulated after ZQ treatment (Table 4). Finally, collagen fibrils are stabilized by crosslinking depending on enzymes of LOX family in the extracellular space, and the collagen fibril is a major component of ECM. Similarly, the levels of LOX mRNA were also upregulated in the ZQ group (Table 4). The above evidence illustrates a complex mechanism of ZQ supplementation in promoting collagen biosynthesis. Moreover, ACSL4, SREBF1, FABP2, LEPR, and MMP3 were enriched in lipid biosynthesis. Transcriptomic analysis of the liver tissue indicated that dietary supplementation with ZQ can affect growth by influencing protein synthesis and fat metabolism.

### 3.5. Transcriptomic Analysis of Skeletal Muscle

**Figure 7:**
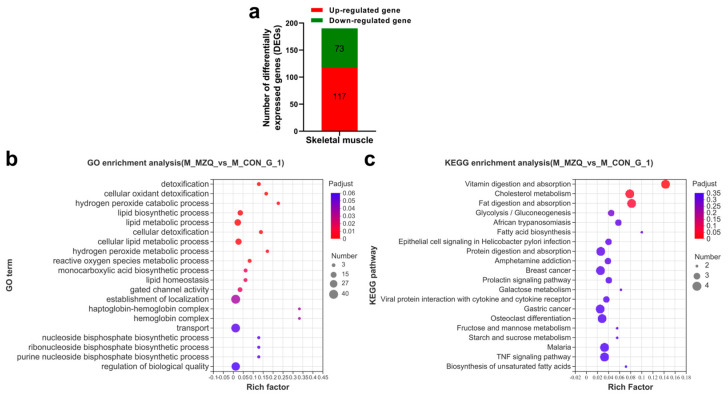
Transcriptomic analysis of skeletal muscle. (**a**) DEGs between the ZQ-1 g and control. (**b**) Bubble plot of the enriched GO terms of skeletal muscle. (**c**) Bubble plot of the enriched KEGG pathways of skeletal muscle.

Meanwhile, the transcriptomic analysis of the skeletal muscle tissue yielded 190 DEGs of which 117 were upregulated and 73 downregulated (Figure 7a, Table S5). The KEGG enrichment analysis indicated that the terms are highly enriched, such as fat digestion and absorption, fatty acid biosynthesis, and protein digestion and absorption (Figure 7c, Table S9). Likewise, the GO enrichment analysis of the DEGs also identified significant enrichment of the genes associated with the lipid biosynthetic process (Figure 7b, Table S8).

**Figure 8:**
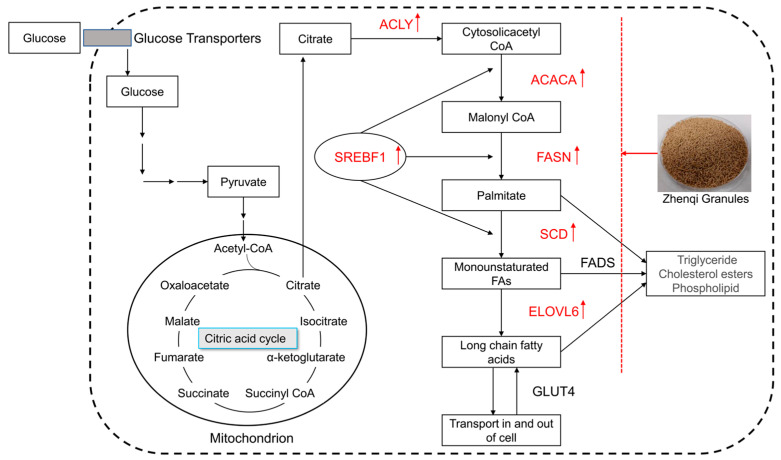
The process of de novo lipogenesis. Note: SREBF1, sterol regulatory element binding transcription factor 1; ACLY, ATP citrate lyase; ACACA, acetyl-CoA carboxylase alpha; FASN, fatty acid synthase; SCD, stearoyl-CoA desaturase; ELOVL6, ELOVL fatty acid elongase 6; FADS, fatty acid desaturase; GLUT4, glucose transporter 4.

In lipogenesis, upon glucose entry into the cell, it is converted into citrate through the tricarboxylic acid (TCA) cycle (Figure 8). Cytosolic acetyl-CoA is generated primarily from citrate by ACLY. Next, acetyl CoA is carboxylated into malonyl-CoA by ACACA, and malonyl CoA is subsequently converted to palmitate by FASN. Thereafter, SCD and ELOVL6 are responsible for creating long-chain fatty acids. The free FA is an essential substrate for the synthesis of TG, phospholipid (PL), and cholesterol esters (CE) [35,36]. Therefore, ACACA plays a critical role in cellular energy storage and lipid synthesis, SCD is essential for porcine adipocyte differentiation and the FASN gene is a promising marker for subcutaneous fat tissue accumulation [37]. The enrichment analysis results of skeletal muscle tissues showed that the mRNA expression of ACLY, ACACA, FASN, SCD, and ELOVL6 were upregulated in pigs supplemented with 1 g/kg of ZQ (Table 5). In addition, the mRNA levels of COL22A1 and SLC36A2, which are closely related to protein digestion and absorption were upregulated in the skeletal muscle of ZQ-1 g pigs (Table 5). Transcriptomic analysis of skeletal muscle (longissimus dorsi) tissues indicated that dietary supplementation with ZQ could affect the de novo lipogenesis in the cell.

## 4. Discussion

Developing nonantibiotic livestock growth promoters attracts intensive research interests in the post-antibiotic era. Traditional Chinese medicine is an important substitute for antibiotics with great development potential. Our preliminary results suggested that ZQ could significantly reduce the F/G of finishing pig after administration for 30 days under field conditions (20 pigs per group), and the F/G of ZQ-1 g/kg was the lowest [32]. In this study, we investigated the growth-promoting efficacy of ZQ in pigs in a more standard experimental condition, and further explored the possible mechanisms by association analysis between growth-promoting traits and transcriptomics data.

Our results showed that, after 30 days administration of ZQ at 1 g/kg or 2 g/kg, the body weight did not show significant difference compared with the control group. It could be because the pigs are in the stage transition from nursery to finishing. At the nursery stage, which is from the weaning stage to 10 weeks and body weight of less than 30 kg [38], energy is mainly used to eliminate negative effects caused by weaning stress and to complete the immune system. Thus, the growth of the nursery pigs is slow [39,40]. Therefore, we continued to monitor the growth for an extra month. On Day 62, the ZQ-1 g group showed a significantly higher body weight and lower F/G compared with the control group. The slaughter performance further revealed higher carcass weight, average back fat thickness, and fat meat percentage. It is worth mentioning that, at the early stage of the experiment, two pigs in the ZQ-1 g group showed obvious stagnant growth, which were then culled. The reason of the stagnant growth was not yet known. Moreover, we performed the slaughter performance measurement at Day 62 (115 days old), since, at this time point, statistical growth difference was observed, and we wanted to carry out the transcriptomic analysis to determine the underlying mechanism of growth promotion. However, it was still a bit early to end the experiment. Normally, slaughter was carried out at 5.5 to 6 months.

This study also found that both doses of ZQ significantly increased the ADG compared with that of the control group. However, the supplementation of ZQ at 2 g/kg did not reveal a better effect on growth promotion than the supplementation at 1 g/kg. Therefore, two-fold supplementation level is not needed when used in practice. This is probably due to the interactions of complex components from TCM [41]. Similar results have been reported in other studies. For example, it was reported that no dose-dependent effect was observed for *Phyllanthus amarus* (PA) extract in protecting the rodents from LPS-induced memory impairment [42]. Fang et al. also found that the serum concentrations of Xiaoyao Pills at a concentration of 8% did not give better results than at 4% [43].

The growth of bone and skeletal muscle, and fat deposition result in increase of body weight of livestock [44]. The results of our study showed that bone growth was not the key factor contributing to the increase in pig growth by ZQ treatment. However, ZQ supplemented at 1 g/kg in feed significantly increased the live weight and carcass weight, indicating that ZQ may increase the fat and muscle content of finishing pigs.

Collagen is the most abundant protein in mammalian bodies, making up from 25% to 35% of the whole-body protein content, and plays structural roles in organizing and maintaining mechanical properties and shape [45]. It is the main protein component of connective tissue in the endomysium of skeletal muscles [46,47]. By transcriptome analysis, our results showed that genes involved in collagen biosynthesis-related proteins were upregulated in the liver tissues of ZQ-treated pigs. A previous study found that the Astragaloside IV, a major ingredient of *Astragalus membranaceus*, may promote the angiogenesis and collagen synthesis [48]. Zeng et al. also reported that the extract from *Astragalus membranaceus* had the activity of inducing type I and type III collagen synthesis [49]. In our study, by comparing the transcriptome of the skeletal muscle tissues between the control group and the ZQ-1 g group, it was shown that the mRNA level of COL22A1 was upregulated in the skeletal muscle of ZQ-1 g pigs. It has been reported that COL22A1 may correlate with serum CREA level which is commonly used to assess kidney function [50,51]. CREA is the end product of creatine metabolism in muscle tissue and its level under normal conditions in serum is positively associated with muscle mass [52], while UREA is the final product of protein metabolism [53]. The results of our study showed that the administration of ZQ caused significant decreases in levels of serum CREA while the level of serum UREA was significantly increased (at 62 d). Therefore, we speculated that the administration of ZQ could promote protein deposition in skeletal muscle, which could also decrease the excessive breakdown of muscle tissue proteins.

Backfat thickness is a good indicator of fat deposition [54]. In this study, the average back fat thickness and fat meat percentage of pigs in ZQ-1 g group were both significantly higher than that of the control group. Consistently, the mRNA of ACACA, FASN, ELOVL6, SCD, ACLY was all significantly upregulated by ZQ in longissimus dorsi. This was consistent with that reported by Crespo-Piazuelo et al. [55]. Meanwhile, the expression of transcripts related to lipid biosynthesis (FRZB, ACSL4, SREBF1) was upregulated in the liver of ZQ-1 g group. Of these, Srebf1 is one of the main regulators of de novo lipogenesis in the liver, and its overexpression contributed to lipids accumulation [56]. However, ZQ decreased the LEPR mRNA levels, LEPR is the receptor for leptin, and leptin promotes lipolysis and limits ectopic deposition in nonfatty tissues [57,58,59,60]. Therefore, we give the following conjecture: On the one hand, ZQ promoted fatty acid (FA) synthesis from glucose by upregulating lipogenic genes such as Srebf1, and then, the FA was esterified to TG and stored in the hepatocyte. On the other hand, less mRNA of LEPR and FABPs corresponds with reduced hepatic triglyceride secretion and serum TG and resultant hepatic lipid accumulation [61,62]. This is evidenced by the decrease in both the serum Glu and TG levels of ZQ-1 g group at 62 d. Those mentioned above suggested that the fat deposition was the main factor of growth promotion of ZQ on finishing pigs. Similar results have been reported in other previous research, the dietary supplementation of Chinese wolfberry and astragalus extracts could improve the growth performance and intervene in key genes related to fatty acid metabolism in Tibetan fragrant pigs [63]. In the review article by Cui et al., they summarized the intramuscular fat deposition-promoting effect of Chinese herbal medicines (CHMs), which contain multiple active ingredients, such as polyphenols, flavonoids, polysaccharides [64].

This study not only demonstrates the growth-promoting effects of Zhenqi Granules in finishing pigs but also further illustrates this effect was primarily through promoting skeletal myogenesis and fat deposition. However, there are still some questions that we have not well understood. For instance, GH and IGF-1, two hormones highly correlated to growth and development, showed no differences between any group. Moreover, the active components of ZQ and their mechanisms of action are unclear.

## 5. Conclusions

This study demonstrated that supplementation of 1 g/kg ZQ in the pig diet in the transition of the nursery pigs to the finishing pigs can improve the important parameters of growth performance and slaughter performance in pigs, while increasing the dose by 2 g/kg did not further increase these effects. This growth-promoting effect was primarily through upregulating the mRNA level of structural proteins and lipid-related enzymes, which, in turn, promotes skeletal myogenesis and fat deposition.

## Figures and Tables

**Table 1:** Ingredients and nutrient levels of the experimental diets.

Ingredients	Content (%)
Corn	51.35
Soybean meal	11.80
Soybean	10.00
Flour	7.00
Broken rice	5.00
Fish meal	3.00
Soybean oil	1.20
Vitamins, minerals and amino acids	5.00
Whey powder	2.50
Glucose	2.50
^1^ Nutrient levels	
Crude protein	≥17.00
Crude fiber	≤4.00
Coarse ash	≤7.00
Calcium	0.50–1.00
Total phosphorus	≥0.60
Sodium chloride	0.30–1.00
^2^ Lysine	≥1.25

Note: ^1^ Nutrient levels are analyzed values. ^2^ Lysine level is the total basis.

**Table 2:** Growth performance at the finishing phase.

Group	Initial Body Weight-17 d (kg)	Final Body Weight-62 d (kg)	ADG (kg/d)	ADFI (kg/d)	F/G
control (N = 8)	30.68 ± 2.85	62.38 ± 6.22	0.70 ± 0.11	1.48	2.10
ZQ-1 g (N = 6)	31.00 ± 2.42	71.92 ± 4.72	0.91 ± 0.13 **	1.48	1.63
ZQ-2 g (N = 8)	30.10 ± 5.53	70.08 ± 10.06	0.89 ± 0.11 **	1.589	1.79

Note: N indicates the number of pigs included in each group; ** indicates *p* < 0.01 between the treatment group (ZQ-1 g or ZQ-2 g) and the control group.

**Table 3:** Effects of ZQ on the biochemical indices of blood.

Days of the Experiment	Indexes	Control (N = 8)	ZQ-1 g (N = 6)	ZQ-2 g (N = 8)
30 d	TP (g/L)	62.50 ± 3.12	61.52 ± 2.73	63.91 ± 3.16
	ALB (g/L)	36.99 ± 2.89	36.75 ± 1.52	39.01 ± 4.27
	Glu (mmol/L)	5.00 ± 0.86	5.52 ± 1.45	5.38 ± 0.86
	ALT (U/L)	86.29 ± 12.05	84.85 ± 16.81	101.19 ± 30.22
	AST (U/L)	52.79 ± 15.01	59.15 ± 19.77	45.69 ± 13.31
	TG (mmol/L)	0.58 ± 0.25	0.74 ± 0.24	0.92 ± 0.18 **
	TC (mmol/L)	2.75 ± 0.37	2.86 ± 0.22	2.88 ± 0.40
	CREA (μmol/L)	94.48 ± 11.29	95.03 ± 13.12	95.14 ± 6.66
	UREA (mmol/L)	1.93 ± 0.58	3.10 ± 1.01 *	2.39 ± 0.83
62 d	TP (g/L)	58.89 ± 2.98	59.47 ± 6.46	58.10 ± 3.07
	ALB (g/L)	35.11 ± 3.48	36.68 ± 3.91	36.75 ± 3.69
	Glu (mmol/L)	3.70 ± 0.58	3.43 ± 0.40	2.95 ± 0.49 **
	ALT (U/L)	79.91 ± 14.13	85.38 ± 9.96	82.16 ± 22.69
	AST (U/L)	172.11 ± 128.72	93.68 ± 21.25 *	120.75 ± 57.83
	TG (mmol/L)	0.45 ± 0.12	0.29 ± 0.04 *	0.31 ± 0.11 *
	TC (mmol/L)	2.65 ± 0.24	2.45 ± 0.13	2.49 ± 0.32
	CREA (μmol/L)	116.10 ± 10.51	95.53 ± 9.70 **	103.96 ± 6.90 *
	UREA (mmol/L)	2.70 ± 0.86	4.04 ± 0.69 **	3.91 ± 0.64 **
	ALP (U/L)	266.81 ± 66.94	255.92 ± 27.39	252.11 ± 31.09

Note: TP, total protein; ALB, albumin; Glu, glucose; ALT, alanine transaminase; AST, aspartate aminotransferase; TG, triglyceride; TC, total cholesterol; CREA, creatinine; UREA, carbamide; ALP, alkaline phosphatase. * indicates *p* < 0.05, ** indicates *p* < 0.01 and NS indicates no significant differences (*p* > 0.05).

**Table 4:** DEGs in the liver of the ZQ-1 g pigs in comparison to the control pigs.

Gene ID	Gene Name	Gene Description	Log2-FC
KEGG: Protein digestion and absorption			
ENSSSCG00000038877	SLC36A3	solute carrier family 36 member 3	3.68
ENSSSCG00000033641	COL8A2	collagen type VIII alpha 2 chain	2.41
ENSSSCG00000036135	COL1A1	collagen type I alpha 1 chain	1.57
ENSSSCG00000015326	COL1A2	collagen type I alpha 2 chain	1.21
ENSSSCG00000016034	COL3A1	collagen type III alpha 1 chain	1.16
ENSSSCG00000027331	COL6A3	collagen type VI alpha 3 chain	1.11
ENSSSCG00000005751	COL5A1	collagen type V alpha 1 chain	1.11
ENSSSCG00000005380	COL15A1	collagen type XV alpha 1 chain	1.02
GO: Collagen/Extracellular matrix (ECM)			
ENSSSCG00000017422	FKBP10	FKBP prolyl isomerase 10	1.42
ENSSSCG00000001914	LOXL1	lysyl oxidase like 1	1.37
ENSSSCG00000000681	P3H3	prolyl 3-hydroxylase 3	1.25
ENSSSCG00000026425	ADAMTSL2	ADAMTS like 2	1.24
ENSSSCG00000024043	ADAMTS2	ADAM metallopeptidase with thrombospondin type 1 motif 2	1.21
ENSSSCG00000017082	SPARC	secreted protein acidic and cysteine rich	1.17
ENSSSCG00000014232	LOX	lysyl oxidase	1.15
ENSSSCG00000033608	LOXL2	lysyl oxidase like 2	1.06
ENSSSCG00000016233	SERPINE2	serpin family E member 2	1.05
ENSSSCG00000039468	SERPINH1	serpin family H member 1	1.00
GO: Lipid biosynthesis			
ENSSSCG00000012583	ACSL4	acyl-CoA synthetase long chain family member 4	1.25
ENSSSCG00000033626	SREBF1	sterol regulatory element binding transcription factor 1	1.01
ENSSSCG00000037272	FABP2	fatty acid binding protein 2	−1.71
ENSSSCG00000025188	LEPR	leptin receptor	−2.19
ENSSSCG00000014985	MMP3	matrix metallopeptidase 3	−2.91

**Table 5:** DEGs in the skeletal muscle of the ZQ-1 g pigs in comparison to the control pigs.

Gene ID	Gene Name	Gene Description	Log2-FC
KEGG: Protein digestion and absorption			
ENSSSCG00000005938	COL22A1	collagen type XXII alpha 1 chain	2.53
ENSSSCG00000017086	SLC36A2	solute carrier family 36 member 2	2.40
GO BP: Lipid biosynthetic process			
ENSSSCG00000014861	MOGAT2	monoacylglycerol O-acyltransferase 2	4.73
ENSSSCG00000010554	SCD	stearoyl-CoA desaturase	3.91
ENSSSCG00000029944	FASN	fatty acid synthase	2.22
ENSSSCG00000025578	ALDH1A2	aldehyde dehydrogenase 1 family member A2	2.07
ENSSSCG00000036236	ELOVL6	ELOVL fatty acid elongase 6	2.06
ENSSSCG00000040689	APOA4	apolipoprotein A4	2.05
ENSSSCG00000017421	ACLY	ATP citrate lyase	1.47
ENSSSCG00000017694	ACACA	acetyl-CoA carboxylase alpha	1.36
ENSSSCG00000009152	SGMS2	sphingomyelin synthase 2	1.15
ENSSSCG00000010483	PLCE1	phospholipase C epsilon 1	1.06
ENSSSCG00000000436	PIP4K2C	phosphatidylinositol-5-phosphate 4-kinase type 2 gamma	1.02
ENSSSCG00000035539	ST8SIA4	ST8 alpha-N-acetyl-neuraminide alpha-2,8-sialyltransferase 4	−1.13
ENSSSCG00000040581	CISH	cytokine inducible SH2 containing protein	−1.16
ENSSSCG00000032481	DPM2	dolichyl-phosphate mannosyltransferase subunit 2, regulatory	−1.28

## Data Availability

Raw Illumina sequencing data were deposited in NCBI (BioProjectID: PRJNA886104).

## Supplementary Materials

The following supporting information can be downloaded at: https://www.mdpi.com/article/10.3390/ani12243521/s1: Table S1: Day-old average body weight of pigs. Table S2: Growth performance throughout the experimental phases. Table S3: Serum GH and IGF-I. Table S4: DEGs of the liver. Table S5: DEGs of the skeletal muscle. Table S6: Liver GO enrichment analysis. Table S7: Liver KEGG enrichment analysis. Table S8: Skeletal muscle GO enrichment analysis. Table S9: Skeletal muscle KEGG enrichment analysis.
