# Supplementary material for: Growth-Promoting Effects of Zhenqi Granules on Finishing Pigs
Source: Animals (Basel). 2022 Dec 13;12(24):3521. doi: 10.3390/ani12243521 (PMC9774107; doi:10.3390/ani12243521)
Supplement: Supplementary file 1 [file animals-12-03521-s001.zip › animals-2032056-supplementary/Table S1-S9/Tables S1-S3.pdf]

Table S1. Day-old average body weight of pig.

Groups	0 d	10 d	17 d	30 d	43 d	57 d	62 d
Control(N=8)	17.79±1.70	25.68±2.42	30.68±2.85	40.08±3.71	49.88±5.34	59.50±6.22	62.38±6.22
ZQ-1g(N=6)	19.23±1.72	27.43±2.20	31.00±2.42	41.75±3.03	53.17±4.73	66.85±4.52	71.92±4.72**
ZQ-2g(N=8)	17.90±3.19	25.023±4.71	30.10±5.53	40.16±6.78	52.09±7.98	65.36±9.50	70.08±10.06

Table S2. Growth performance throughout experimental phases.

Groups	Initial body weight-0 d (kg)	Final body weight-62 d (kg)	ADG (kg/d)	ADFI (kg/d)	F/G
Control(N=8)	17.79±1.70	62.38±6.22	0.59±0.09	1.43	2.41
ZQ-1g(N=6)	19.23±1.72	71.92±4.72*	0.72±0.08*	1.38	1.92
ZQ-2g(N=8)	17.90±3.19	70.08±10.06	0.73±0.09**	1.47	2.03

Table S3. Serum GH and IGF-I.

	Groups	0 d	17 d	30 d	43 d	57 d	62 d
GH (ng/mL)	Control	5.97±4.18	4.96±4.09	8.75±4.28	16.02±5.76	18.44±3.95	18.55±6.13
	ZQ-1g	6.62±5.61	5.99±2.72	7.93±7.74	16.55±8.06	17.77±2.94	18.57±5.05
	ZQ-2g	7.74±4.06	5.51±3.35	11.75±5.56	15.09±5.89	17.45±5.88	18.39±2.78
IGF-I (ng/mL)	Control	19.29±15.51	76.88±96.34	30.21±23.50	42.26±33.60	23.55±21.55	35.16±24.79
	ZQ-1g	28.55±28.54	63.64±67.70	68.44±50.35	52.88±30.79	16.67±14.57	29.89±15.65
	ZQ-2g	15.71±9.17	97.59±110.49	47.51±34.99	38.29±10.56	25.94±21.12	27.76±18.95

Note: GH, growth hormone; IGF-1, insulin like growth factor-1.
